# Ouabain Suppresses IL-6/STAT3 Signaling and Promotes Cytokine Secretion in Cultured Skeletal Muscle Cells

**DOI:** 10.3389/fphys.2020.566584

**Published:** 2020-09-25

**Authors:** Sergej Pirkmajer, Katja Bezjak, Urška Matkovič, Klemen Dolinar, Lake Q. Jiang, Katarina Miš, Katarina Gros, Kseniya Milovanova, Katja Perdan Pirkmajer, Tomaž Marš, Leonid Kapilevich, Alexander V. Chibalin

**Affiliations:** ^1^Institute of Pathophysiology, Faculty of Medicine, University of Ljubljana, Ljubljana, Slovenia; ^2^Integrative Physiology, Department of Molecular Medicine and Surgery, Karolinska Institutet, Stockholm, Sweden; ^3^Department of Sports and Health Tourism, Sports Physiology and Medicine, National Research Tomsk State University, Tomsk, Russia; ^4^Department of Rheumatology, University Medical Centre Ljubljana, Ljubljana, Slovenia; ^5^Department of Internal Medicine, Faculty of Medicine, University of Ljubljana, Ljubljana, Slovenia; ^6^Central Scientific Laboratory, Siberian State Medical University, Tomsk, Russia

**Keywords:** Ouabain, marinobufagenin, Na, K-ATPase, cytokines, skeletal muscle, IL-6

## Abstract

The cardiotonic steroids (CTS), such as ouabain and marinobufagenin, are thought to be adrenocortical hormones secreted during exercise and the stress response. The catalytic α-subunit of Na,K-ATPase (NKA) is a CTS receptor, whose largest pool is located in skeletal muscles, indicating that muscles are a major target for CTS. Skeletal muscles contribute to adaptations to exercise by secreting interleukin-6 (IL-6) and plethora of other cytokines, which exert paracrine and endocrine effects in muscles and non-muscle tissues. Here, we determined whether ouabain, a prototypical CTS, modulates IL-6 signaling and secretion in the cultured human skeletal muscle cells. Ouabain (2.5–50 nM) suppressed the abundance of STAT3, a key transcription factor downstream of the IL-6 receptor, as well as its basal and IL-6-stimulated phosphorylation. Conversely, ouabain (50 nM) increased the phosphorylation of ERK1/2, Akt, p70S6K, and S6 ribosomal protein, indicating activation of the ERK1/2 and the Akt-mTOR pathways. Proteasome inhibitor MG-132 blocked the ouabain-induced suppression of the total STAT3, but did not prevent the dephosphorylation of STAT3. Ouabain (50 nM) suppressed hypoxia-inducible factor-1α (HIF-1α), a modulator of STAT3 signaling, but gene silencing of HIF-1α and/or its partner protein HIF-1β did not mimic effects of ouabain on the phosphorylation of STAT3. Ouabain (50 nM) failed to suppress the phosphorylation of STAT3 and HIF-1α in rat L6 skeletal muscle cells, which express the ouabain-resistant α1-subunit of NKA. We also found that ouabain (100 nM) promoted the secretion of IL-6, IL-8, GM-CSF, and TNF-α from the skeletal muscle cells of healthy subjects, and the secretion of GM-CSF from cells of subjects with the type 2 diabetes. Marinobufagenin (10 nM), another important CTS, did not alter the secretion of these cytokines. In conclusion, our study shows that ouabain suppresses the IL-6 signaling via STAT3, but promotes the secretion of IL-6 and other cytokines, which might represent a negative feedback in the IL-6/STAT3 pathway. Collectively, our results implicate a role for CTS and NKA in regulation of the IL-6 signaling and secretion in skeletal muscle.

## Introduction

The cardiotonic steroids (CTS) are thought to be a family of adrenocortical and/or hypothalamic hormones (Schoner, 2001, 2002; Schoner and Scheiner-Bobis, 2008; Bagrov et al., 2009; Hamlyn and Blaustein, 2016; Blaustein, 2018; Blaustein and Hamlyn, 2020). The endogenous and the exogenous CTS are structurally divided into cardenolides, which contain an unsaturated five-membered lactone ring, and bufadienolides, which contain an unsaturated six-membered lactone ring (Schoner, 2002; Bagrov et al., 2009). The endogenous CTS may include cardenolides, such as ouabain (Hamlyn et al., 1991; Mathews et al., 1991) and/or closely related stereoisomers, and bufadienolides, such as marinobufagenin (Bagrov et al., 1998), telocinobufagin (Komiyama et al., 2005), and bufalin (Kieval et al., 1988; Lichtstein et al., 1993).

Cardiotonic steroids, whose endogenous secretion is believed to be regulated by the sympathoadrenergic system, the adrenocorticotropic hormone, and angiotensin II (Laredo et al., 1995, 1997; Bauer et al., 2005), may have an important role in regulation of Na^+^ homeostasis, arterial blood pressure, as well as the stress and immune responses (Foey et al., 1997; Fedorova et al., 2001; Berendes et al., 2003; Blaustein et al., 2012; Cavalcante-Silva et al., 2017). As estimated by an ouabain immunoassay, plasma concentrations of CTS are markedly increased during exercise (Bauer et al., 2005), but their physiological role under these conditions has not been established. Contracting skeletal muscles secrete interleukin-6 (IL-6) and a plethora of other cytokines (aka myokines), which regulate the immune system and metabolic pathways in skeletal muscle and other metabolic organs, thus contributing to acute and chronic adaptations to exercise (Pedersen and Febbraio, 2012; Egan and Zierath, 2013; Pedersen, 2019). Skeletal muscles are therefore a major source as well as an important site of cytokine action during exercise. Here, we asked whether CTS might alter signaling and the secretion of IL-6 in skeletal muscle cells.

Cardiotonic steroids exert their effects by binding to Na,K-ATPase (NKA), which serves as their primary receptor (Schoner, 2002; Dostanic-Larson et al., 2005; Bagrov et al., 2009; Aperia et al., 2016; Blaustein, 2018), although existence of other receptors cannot be excluded (Askari, 2019b). NKA is an ion pump that maintains ion homeostasis by transporting three Na^+^ ions from and two K^+^ ions into the cell (Post and Jolly, 1957; Skou, 1957, 1965; Sen and Post, 1964). It comprises a catalytic α-subunit (isoforms α1-4), which contains the CTS binding site (Ruoho and Kyte, 1974; Kent et al., 1987; Ogawa et al., 2009; Lingrel, 2010), and a glycoprotein β-subunit (isoforms β1-3 in mammals) (Blanco and Mercer, 1998; Pestov et al., 2007, 2011; Geering, 2008). CTS inhibit transport activity of NKA, thus increasing Na^+^ ([Na^+^]_i_) and decreasing K^+^ ([K^+^]_i_) concentrations in the cell (Schatzmann, 1953; Post and Jolly, 1957; Post et al., 1960; Lamb and McCall, 1972), which leads to complex alterations in transcriptional activity (Klimanova et al., 2017). An increase in [Na^+^]_i_ suppresses Ca^2+^ extrusion via the Na^+^/Ca^2+^ exchanger (NCX), thereby elevating intracellular Ca^2+^ concentrations (Baker et al., 1969; Blaustein and Hamlyn, 2020; Pavlovic, 2020). An increase in [Na^+^]_i_ may also stimulate Na^+^-sensitive signaling pathways (Kuroki et al., 1997), while a decrease in [K^+^]_i_ suppresses protein translation (Orlov et al., 2017; Amarelle et al., 2019; Klimanova et al., 2019).

As well as an ion transporter, NKA is a signal transducer (Xie and Askari, 2002). NKA forms a signaling complex with the epithelial growth factor receptor (EGFR) and Src kinase (Haas et al., 2000; Tian et al., 2006; Bagrov et al., 2009; Pavlovic, 2020). Once bound to NKA, ouabain and some other CTS activate the complex, which stimulates downstream signaling pathways, such as the ERK1/2 pathway (Haas et al., 2000; Xie and Askari, 2002; Kotova et al., 2006a, b). In addition, CTS activate phosphatidylinositol 3-kinase, thus leading to stimulation of the Akt (aka protein kinase B) and mechanistic (aka mammalian) target of rapamycin (mTOR) pathway, which is thought to be particularly important in relation to cardiac hypertrophy (Liu et al., 2007). NKA also interacts with the inositol 1,4,5-trisphosphate receptor via which ouabain triggers oscillations in intracellular concentrations of Ca^2+^ (Aizman et al., 2001; Zhang et al., 2006) and alters activity of multiple protein kinases (Panizza et al., 2019). While all CTS inhibit NKA (Lingrel, 2010), different CTS may have similar, distinct, or even opposing signaling and physiological effects (Manunta et al., 2000; Kotova et al., 2006a; Zulian et al., 2013). Low concentrations of CTS, which only weakly inhibit NKA transport activity, have pronounced signaling effects (Kotova et al., 2006a). CTS-induced activation of signaling pathways via NKA therefore probably does not require significant alterations in [Na^+^]_i_ or [K^+^]_i_ and appears to be at least partially independent of its ion transport activity. NKA with a mutant α1-subunit that pumps but is defective in signal transduction was described (Lai et al., 2013), indirectly supporting the idea that the two functions might be partially dissociated. Nevertheless, alterations in [Na^+^]_i_ or [K^+^]_i_, which arise due to inhibition of NKA, are likely an important mechanism by which CTS modulate intracellular signaling (Klimanova et al., 2017, 2019; Orlov et al., 2017; Askari, 2019a).

Skeletal muscles contain a major pool of NKA in the body (Clausen, 1996, 2010) and are therefore an important target for CTS (Glantz et al., 1976; Kjeldsen et al., 1985; Harashima et al., 1988). However, only limited data regarding the role of CTS in skeletal muscle is available. In mice, infusion of the CTS-binding antibody increases transport activity of NKA during muscle contractions, which demonstrates that CTS might be involved in acute regulation of NKA (Radzyukevich et al., 2009). In cultured skeletal muscle cells and isolated skeletal muscle, ouabain stimulates the glycogen synthesis (Clausen, 1965; Kotova et al., 2006a, b), suggesting a role for CTS and NKA in regulation of skeletal muscle metabolism (Pirkmajer and Chibalin, 2016). Interestingly, recent data suggest that circulating ouabain may regulate NKA in skeletal muscle and oppose depolarization of the sarcolemma due to muscle disuse or exposure to lipopolysaccharide (Kravtsova et al., 2020). Clearly, the role of CTS in skeletal muscle needs to be dissected in more detail. Using primary human skeletal muscle cells we examined whether and how CTS modulate the IL-6/STAT3 signaling. We also determined whether ouabain and marinobufagenin modulate the secretion of IL-6 and other muscle-derived cytokines.

## Materials and Methods

### Materials and Antibodies

Cell culture flasks and plates were from Sarstedt or TPP. Advanced MEM, GlutaMAX, MEM vitamin solution, DMEM, fetal bovine serum (FBS), trypsin-EDTA, pen strep (5000 units/ml of penicillin and 5000 μg/ml of streptomycin), fungizone (250 μg/ml of amphotericin B), gentamicin (10 mg/ml), Pierce BCA Protein Assay Kit, Pierce Enhanced Chemiluminescence (ECL) Western Blotting Substrate, High-Capacity cDNA Reverse Transcription Kit, TaqMan Universal Master Mix and TaqMan gene expression assays for IL-6 (Hs00174131_m1), HIF1A (Hs00153153_m1), ARNT/HIF1β (Hs00231048_m1), ATP1A1 (Hs00167556_m1), ATP1A2 (Hs00265131_m1), ATP1A3 (Hs00958036_m1), and actin-β (ACTB, Hs99999903_m1), PPIA (Hs99999904_m1), 18S rRNA (Hs99999901_s1), rat ATP1A1 (Rn01533986_m1), rat ATP1A2 (Rn00560789_m1), rat ATP1A3 (Rn00560813_m1) and rat ACTB (4352931E) were from Thermo Fisher Scientific. PCR plates, PCR plate sealing films, 4–12% Criterion XT Bis-Tris polyacrylamide gels, XT MES electrophoresis buffer and goat anti-rabbit or anti-mouse IgG - horseradish peroxidase conjugate were from Bio-Rad. Amersham ECL Full-Range Rainbow Molecular Weight Markers were from GE Healthcare Life Sciences. Polyvinylidene fluoride (PVDF) membrane was from Merck Millipore. CP-BU NEW X-ray films were form AGFA HealthCare. RNeasy Plus Mini Kit was from Qiagen. E.Z.N.A. HP Total RNA Kit was from Omega Bio-Tek. Recombinant human IL-6 was from PeproTech and antibody against IL-6 receptor was from Roche (tocilizumab, RoActemra). Proteasome inhibitor MG-132, ouabain octahydrate, puromycin, and all other reagents, unless otherwise specified, were from Sigma-Aldrich (Merck). Marinobufagenin was a kind gift from Dr. Alexei Bagrov (National Institute of Aging, NIH, Baltimore).

Target proteins were detected using primary antibodies against phospho-STAT3 (Tyr705) (Cell Signaling #9145), STAT3 (Cell Signaling #4904), phospho-ERK1/2 (Thr202/Tyr204) (Cell Signaling #4370 or #9101), ERK1/2 (Cell Signaling #4695), phospho-4E-BP1 (Thr37/46) (Cell Signaling #2855), 4E-BP1 (Cell Signaling #9644), α1-subunit of NKA (Upstate #05-369), phospho-Src (Tyr527) (Cell Signaling #2105), phospho-S6 ribosomal protein (Ser235/236) (Cell Signaling #2211), S6 ribosomal protein (Cell Signaling #2217), phospho-p70S6K (Thr389) (Cell Signaling #9205), phospho-Akt (Ser473) (Cell Signaling #4060), total Akt (Cell Signaling #4691), HIF-1α (Novus Biologicals #NB100-449), HIF-1β/ARNT (Cell Signaling #5537).

### Ethical Approvals

Preparation of primary human skeletal muscle cells and experimental procedures involving these cells were approved by the Republic of Slovenia National Medical Ethics Committee (ethical approval no. 71/05/12 and 0120-698/2017/4) or Ethics Committee at Karolinska Institutet (ethical approval no. DNR 2006/225-31/1).

### Primary Human Skeletal Muscle Cell Cultures

Human skeletal muscle cells were prepared from samples of the *semitendinosus* muscle (Procedure 1) as described (Dolinar et al., 2018) for all experiments excepting those conducted to compare effects of ouabain and marinobufagenin on the cytokine secretion. Briefly, according to the Procedure 1 satellite cells were released by trypsinization of muscle samples at 37°C. Primary cultures were grown at 37°C in humidified air with 5% (v/v) CO_2_ in Advanced MEM [with 1% (v/v) GlutaMAX, 1% (v/v) MEM vitamin solution], 10% (v/v) FBS, 0.3% (v/v) fungizone, and 0.15% (v/v) gentamicin. Skeletal muscle cells were purified with MACS CD56 MicroBeads (Miltenyi Biotec) before reaching confluence. To differentiate myoblasts into myotubes, myoblasts were switched to Advanced MEM with 2% (v/v) FBS for 7–9 days. For estimation of the cytokine secretion we used skeletal muscle cells prepared from samples of the *vastus lateralis* muscle (Procedure 2) as described (Kotova et al., 2006a). Characteristics of healthy (Control) subjects and subjects with the type 2 diabetes (Table 1) were previously reported for a larger cohort (Jiang et al., 2013). According to the Procedure 2 satellite cells were released from muscle tissue by incubating muscle samples in Trypsin-EDTA at 37°C. To increase the purity of the myogenic fraction, the released cells were plated for 1 h in the Petri plates. Supernatants were used for further culturing. Cells were subsequently maintained in DMEM/F12 supplemented with 20% (v/v) FBS, 1% (v/v) pen strep and 1% (v/v) fungizone at 37°C in humidified air with 7% (v/v) CO_2_. Myoblasts were differentiated into myotubes for 7–8 days in DMEM [2% (v/v) FBS, 1% (v/v) pen strep and 1% (v/v) fungizone].

**TABLE 1:** Baseline characteristics of the donors of skeletal muscle cells that were used for cytokine measurements.

Clinical characteristics	NGT	T2D
*n* (male)	5	4
Age (years)	63.0 ± 3.5	59.3 ± 6.6
BMI (kg/m^2^)	27.0 ± 1.7	27.8 ± 2.5
Waist (cm)	102.6 ± 7.3	100.3 ± 6.6
Fasting plasma glucose (mM)	5.0 ± 0.2	7.4 ± 0.3***
2-h plasma glucose (mM)	6.0 ± 0.5	16.2 ± 0.7***
HbA_1__c_ (%)	4.6 ± 0.2	5.9 ± 0.8**
Total cholesterol (mM)	5.7 ± 0.8	4.7 ± 0.5
LDL (mM)	3.2 ± 1.1	2.9 ± 0.2
HDL (mM)	1.8 ± 0.7	1.4 ± 0.3
Triglyceride (mM)	1.5 ± 1.1	1.0 ± 0.1
SBP (mmHg)	143.0 ± 6.7	153.8 ± 16.5
DBP (mmHg)	91.0 ± 4.2	85.0 ± 0*
Hemoglobin (g/L)	158 ± 8	149 ± 7

DBP, diastolic blood pressure; NGT, normal glucose tolerance; SBP, systolic blood pressure; T2D, type 2 diabetes. *p < 0.05; **p < 0.01; ***p < 0.00001 vs. NGT.

### L6 Skeletal Muscle Cells

L6 cells were obtained from ATCC and cultured as described (Dolinar et al., 2018). Briefly, L6 cells were cultured in MEMα supplemented with 10% (v/v) FBS, 1% (v/v) pen strep, and 0.3% (v/v) fungizone. All experiments with L6 muscle cells were performed on the differentiated myotubes. To differentiate myoblasts into myotubes, myoblasts were grown in the presence of 10% (v/v) FBS until they were almost confluent and then for additional 7–9 days in MEMα with 2% (v/v) FBS.

### Gene Silencing of HIF-1α and HIF-1β in Human Myoblasts

The primary human skeletal muscle cells were seeded into 6-well (for immunoblotting) or 12-well (for qPCR) plates and cultured overnight in Advanced MEM with 10% (v/v) FBS and without antibiotics and antimycotics. The transfections were performed using lipofectamine 2000 reagent (Thermo Fisher Scientific) according to the manufacturer’s protocol. Lipofectamine and siRNA were separately diluted in Opti-MEM (Thermo Fisher Scientific) and combined just before addition to the cell culture medium. Final concentrations of siRNA in the medium were: 5 nM siRNA against HIF-1α (J-004018–10 selected as the most effective from the ON-TARGETplus SMARTpool set) and/or 10 nM of each siRNA (J-007207-06, -07, -08, and -09) from the ON-TARGETplus SMARTpool set against HIF-1β, or scrambled siRNA (ON-TARGETplus Non-targeting Pool, D-001810-10-20) at 5 nM (siSCR1) and 45 nM (siSCR2) concentration (all from Dharmacon Horizon Discovery). Final concentration of lipofectamine was 2‰ (v/v). After 24-h incubation in the presence of siRNA, the cell medium was replaced with the complete growth medium. Experiments were performed 24 h after the medium replacement.

### Immunoblotting

Immunoblotting was performed as described (Dolinar et al., 2018). Briefly, at the end of the experiment, cells were washed with the ice-cold phosphate-buffered saline (PBS: 137 mM NaCl, 2.7 mM KCl, 10 mM Na_2_HPO_4_, 1.8 mM KH_2_PO_4_, pH 7.4) and lysed in the Laemmli buffer [62.5 mM Tris-HCl (pH 6.8), 2% (w/v) sodium dodecyl sulfate (SDS), 10% (w/v) glycerol, 5% (v/v) 2-mercaptoethanol, 0.002% (w/v) bromophenol blue]. Proteins were resolved with SDS-PAGE (4–12% polyacrylamide gels) and transferred to the PVDF membrane with wet electrotransfer. After the transfer, membranes were stained with Ponceau S [0.1% (w/v) in 5% (v/v) acetic acid] to evaluate uniformity of sample loading and transfer. Membranes were then blocked with 7.5% (w/v) dry milk in the Tris-buffered saline with Tween 20 [TBST: 20 mM Tris, 150 mM NaCl, 0.02% (v/v) Tween 20, pH 7.5] for 1 h at room temperature. After blocking, membranes were incubated with a primary antibody in the primary antibody buffer [20 mM Tris, 150 mM NaCl, pH 7.5, 0.1% (w/v) BSA and 0.1% (w/v) sodium azide] overnight at 4°C and then with the secondary antibody-horseradish peroxidase conjugate in TBST with 5% (w/v) dry milk for 1 h at room temperature. Finally, membranes were incubated with ECL reagent and then immunolabeled proteins were visualized on the X-ray films. Films were scanned with GS-800 Densitometer (Bio-Rad) and analyzed with Quantity One 1-D Analysis Software (Bio-Rad). Intensities of individual bands were expressed in arbitrary units relative to the total intensity of all the bands.

### Quantitative Real-Time Polymerase Chain Reaction

Total RNA was extracted with RNeasy Plus Mini Kit or E.Z.N.A. HP Total RNA Kit and reverse transcribed to cDNA with High-Capacity cDNA Reverse Transcription Kit. The quantitative real-time polymerase chain reaction (qPCR) was performed on 7500 Real-Time PCR System (Applied Biosystems, Thermo Fisher Scientific) using TaqMan Universal Master Mix and TaqMan gene expression assays. The endogenous controls (reference genes) were actin-β (ACTB), cyclophilin (PPIA), and 18S rRNA. ACTB was the endogenous control for the gene silencing (of HIF-1α and/or HIF-1β) experiment. To estimate effects of ouabain on IL-6 and NKAα1 mRNA the geometric mean of three endogenous controls (ACTB mRNA, PPIA mRNA, and 18S rRNA) was used for normalization. Efficiency of PCR was estimated with the LinRegPCR software (Ramakers et al., 2003; Ruijter et al., 2009).

### Cytokine Measurements

Cultured myotubes were treated with 100 nM ouabain or 10 nM marinobufagenin for 16 h in serum-free DMEM. Once the 16-h treatment with ouabain and marinobufagenin was completed, media samples were collected and stored at −20°C. Measurements of cytokines were performed using High Sensitivity Human Cytokine Multiplex Immunoassay (Millipore) on Bio-Plex Multiplex System (BioRad) according to the manufacturer’s instructions. The amount of secreted cytokines was normalized to the total cellular protein content as estimated with the BCA protein assay.

### Statistical Analysis

Data are presented as means with standard error of the mean (SEM) or the standard deviation (SD) as indicated. In all experiments with human skeletal muscle cells, *n* refers to the number of donors (primary cultures obtained from different donors). Statistical analysis was performed with GraphPad Prism 6 and 8 (GraphPad Software) using ANOVA with Dunnett’s or Bonferroni’s test, Kruskal–Wallis and Dunn’s test, or Wilcoxon signed-rank test. The difference between the groups was considered statistically significant when *p* < 0.05.

## Results

### Ouabain Suppresses IL-6 Signaling in Cultured Human Myotubes by Reducing the Abundance and the Phosphorylation of STAT3

**FIGURE 1:**
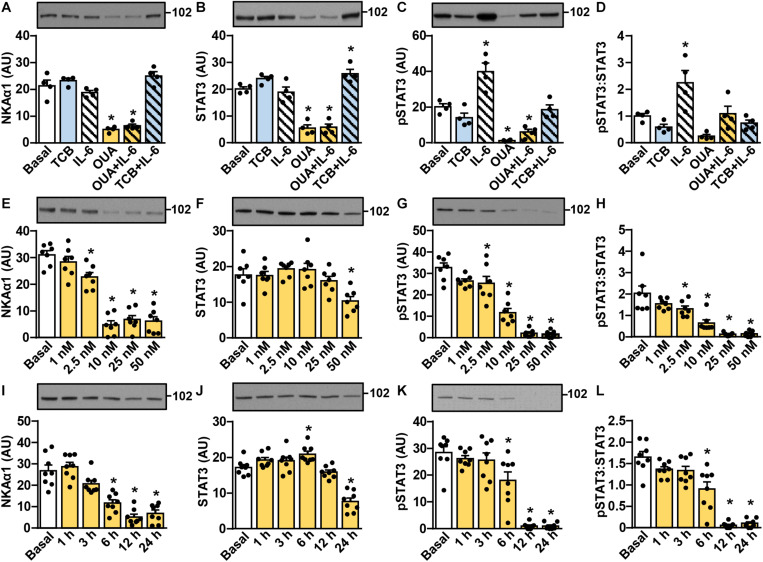
Ouabain suppresses IL-6 signaling in cultured human myotubes by reducing the abundance and the phosphorylation of STAT3. **(A–D)** Human myotubes were treated with or without 50 nM ouabain (OUA) in Advanced MEM with 2% FBS for 20 h and in serum-free Advanced MEM for 4 h. Cells were treated with tocilizumab (100 μg/ml, TCB) during the last 4 h and with interleukin-6 (50 ng/ml, IL-6) during the last 15 min. **(E–L)** Human myotubes were treated in serum-free Advanced MEM with different concentrations of OUA for 24 h **(E–H)** or with 50 nM OUA for 1–24 h **(I–L)**. **(A,E,I)** α1-subunit of Na,K-ATPase (NKAα1), **(B,F,J)** the total STAT3 and **(C,G,K)** phospho-STAT3 (Tyr705) were measured by immunoblotting. Graphs in panels **(D,H,L)** show the phospho-STAT3:STAT3 ratio. Results are means with SEM (*n* = 4–8). *p < 0.05 vs. Basal.

STAT3, a transcription factor, is activated (phosphorylated) in skeletal muscle by IL-6 and exercise (Trenerry et al., 2007; Pedersen and Febbraio, 2008). To determine whether ouabain modulates IL-6 signaling in skeletal muscle cells, we treated myotubes with 50 nM ouabain for 20 h in Advanced MEM, supplemented with 2% FBS. This was followed by a 4-h treatment with 50 nM ouabain and/or 100 μg/ml tocilizumab in serum-free Advanced MEM. Myotubes were stimulated with 50 ng/ml IL-6 (Figures 1A–D) during the last 15 min. Ouabain decreased the abundance of the α1-subunit of NKA (NKAα1) (Figure 1A) and STAT3 (Figure 1B). The basal and the IL-6-stimulated phosphorylation of STAT3 (Tyr705) were also reduced by ouabain (Figures 1C,D). Tocilizumab, an antibody against the α-subunit (IL-6Rα) of the oligomeric IL-6 receptor (IL-6Rα/gp130), blocked the IL-6-stimulated phosphorylation of STAT3 without significantly altering its basal phosphorylation (Figures 1C,D) or the abundance of STAT3 (Figure 1B).

In humans intravenous application of ouabain results in peak plasma concentrations in the ∼10 nM range, while chronic steady-state concentrations are below 1 nM (Selden and Smith, 1972; Lewis et al., 1994; Pidgeon et al., 1994). To determine the concentration dependency of ouabain effects, myotubes were treated with 1–50 nM ouabain for 24 h in serum-free Advanced MEM (Figures 1E–H). While low concentrations of ouabain (1 and 2.5 nM) already tended to reduce the level of NKAα1, 10 nM ouabain reduced it by more than 80% (Figure 1E). The phosphorylation of STAT3 was reduced in a dose-dependent manner by 2.5–50 nM ouabain (Figures 1F,G). The abundance of STAT3 was reduced only by 50 nM ouabain. The phospho-STAT3:STAT3 ratio was reduced already by 2.5–25 nM ouabain (Figure 1H), indicating that lower concentrations of ouabain are required to suppress the phosphorylation than to reduce the abundance of STAT3.

After a single intravenous dose, ouabain displays a rapid initial decline, followed by a late phase of slow elimination, which has a half-life of more than ∼20 h (Selden and Smith, 1972). To determine the time course of ouabain effects, human myotubes were treated with 50 nM ouabain for 1–24 h in serum-free Advanced MEM (Figures 1I–L). The abundance of NKAα1 was significantly reduced after 6 h and reached its lowest level at 12 h, which was maintained up to 24 h (Figure 1I). The total STAT3 was relatively stable for the first 12 h of treatment, but was reduced at 24 h (Figure 1J), consistent with previous experiments (Figures 1B,F). The phosphorylation of STAT3 was also relatively stable for the first 3 h of treatment, but was almost totally suppressed at 12 and 24 h of ouabain treatment (Figure 1K). As estimated by the phospho-STAT3:STAT3 ratio (Figure 1L), these results again indicate that ouabain reduces the phosphorylation before reducing the abundance of STAT3, which suggests that ouabain affects STAT3 signaling via two mechanisms.

### Ouabain Stimulates Activation of the ERK1/2 Pathway in Cultured Human Myotubes in a Time- and Concentration-Dependent Manner

**FIGURE 2:**
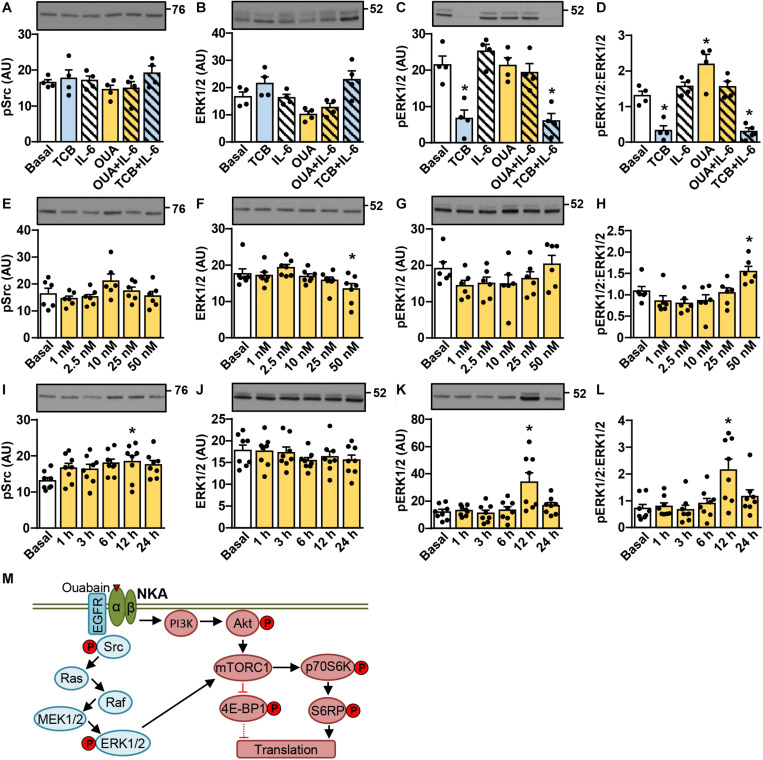
Ouabain stimulates activation of the ERK1/2 pathway in cultured human myotubes in a time- and concentration-dependent manner. **(A–D)** Human myotubes were treated with or without 50 nM ouabain (OUA) in Advanced MEM with 2% FBS for 20 h and in serum-free Advanced MEM for 4 h. Cells were treated with tocilizumab (100 μg/ml, TCB) during the last 4 h and with interleukin-6 (50 ng/ml, IL-6) during the last 15 min. **(E–L)** Human myotubes were treated in serum-free Advanced MEM with different concentrations of OUA for 24 h **(E–H)** or with 50 nM OUA for 1–24 h **(I–L)**. **(A,E,I)** phospho-Src (Tyr527), **(B,F,J)** the total ERK1/2 and **(C,G,K)** phospho-ERK1/2 (Thr202/Tyr204) were measured by immunoblotting. Graphs in panels **(D,H,L)** show the phospho-ERK1/2:ERK1/2 ratio. Results are means with SEM (*n* = 4–8). *p < 0.05 vs. Basal. **(M)** Schematic overview of ouabain signaling via the Src-ERK1/2 pathway and the phosphatidylinositol 3-kinase (PI3K)/Akt/mTOR pathway. ERK1/2 is a known upstream regulator of the mTOR complex 1 (mTORC1), indicating crosstalk between the two pathways. Phosphoproteins that were assessed in this study are indicated by P.

Ouabain is thought to activate the signaling complex comprising NKA, EGFR, and Src, which leads to activation of the ERK1/2 pathway (Xie and Askari, 2002; Kotova et al., 2006a). We estimated activity of this pathway by measuring the inhibitory phosphorylation of Src (Tyr527) and the activating phosphorylation of ERK1/2 (Thr202/Tyr204) (Figure 2). Experiments were the same as those described in Figure 1. The phosphorylation of Src was increased by the 12-h treatment with 50 nM ouabain (Figure 2I), but was otherwise unresponsive to all treatments. Ouabain tended to increase the phosphorylation of ERK1/2 and/or the phospho-ERK1/2:ERK1/2 ratio. The effect was most pronounced at 12 h (Figures 2K,L), but was also observed at 24 h (Figures 2D,H). Tocilizumab, which blocks IL-6Rα, markedly suppressed the phosphorylation of ERK1/2 (Figures 2C,D), indicating that the IL-6Rα/gp130 receptor complex is active under the basal conditions. While these experiments showed that ouabain activated the ERK1/2 pathway (schematically presented in Figure 2M), its response was delayed. However, if we treated myotubes with 100 nM ouabain (a cardenolide) and 10 nM marinobufagenin (a bufadienolide), which were previously shown to inhibit NKA transport activity to a similar degree (Kotova et al., 2006a), ERK1/2 response was observed between 2–6 h (data not shown).

### Ouabain Stimulates Activation of the Akt-mTOR Pathway in Cultured Human Myotubes in a Time- and Concentration-Dependent Manner

**FIGURE 3:**
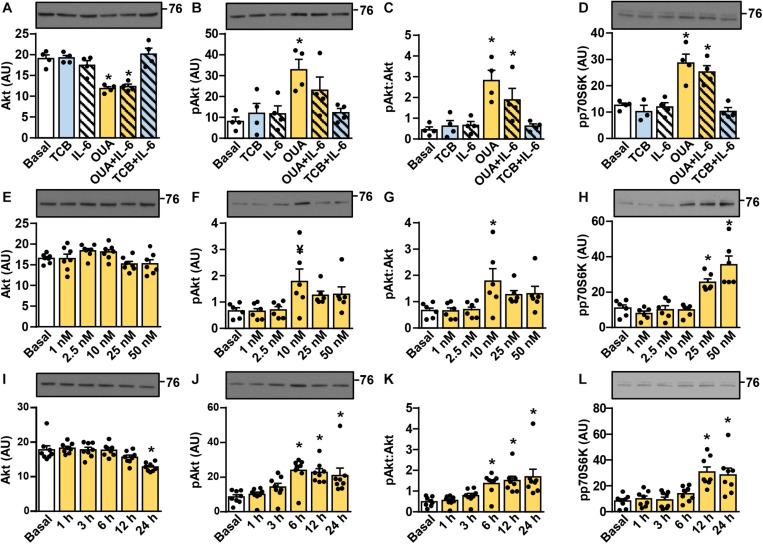
Ouabain stimulates the phosphorylation of Akt and p70S6K in cultured human myotubes. **(A–D)** Human myotubes were treated with or without 50 nM ouabain (OUA) in Advanced MEM with 2% FBS for 20 h and in serum-free Advanced MEM for 4 h. Cells were treated with tocilizumab (100 μg/ml, TCB) during the last 4 h and with interleukin-6 (50 ng/ml, IL-6) during the last 15 min. **(E–L)** Human myotubes were treated in serum-free Advanced MEM with different concentrations of OUA for 24 h **(E–H)** or with 50 nM OUA for 1–24 h **(I–L)**. **(A,E,I)** The total Akt, **(B,F,J)** phospho-Akt (Ser473), and **(D,H,L)** phospho-p70S6K (Thr389) were measured by immunoblotting. Graphs in panels **(C,G,K)** show the phospho-Akt:Akt ratio. Results are means with SEM (*n* = 4–8). *p < 0.05 vs. Basal, ^¥^*p* = 0.058.

**FIGURE 4:**
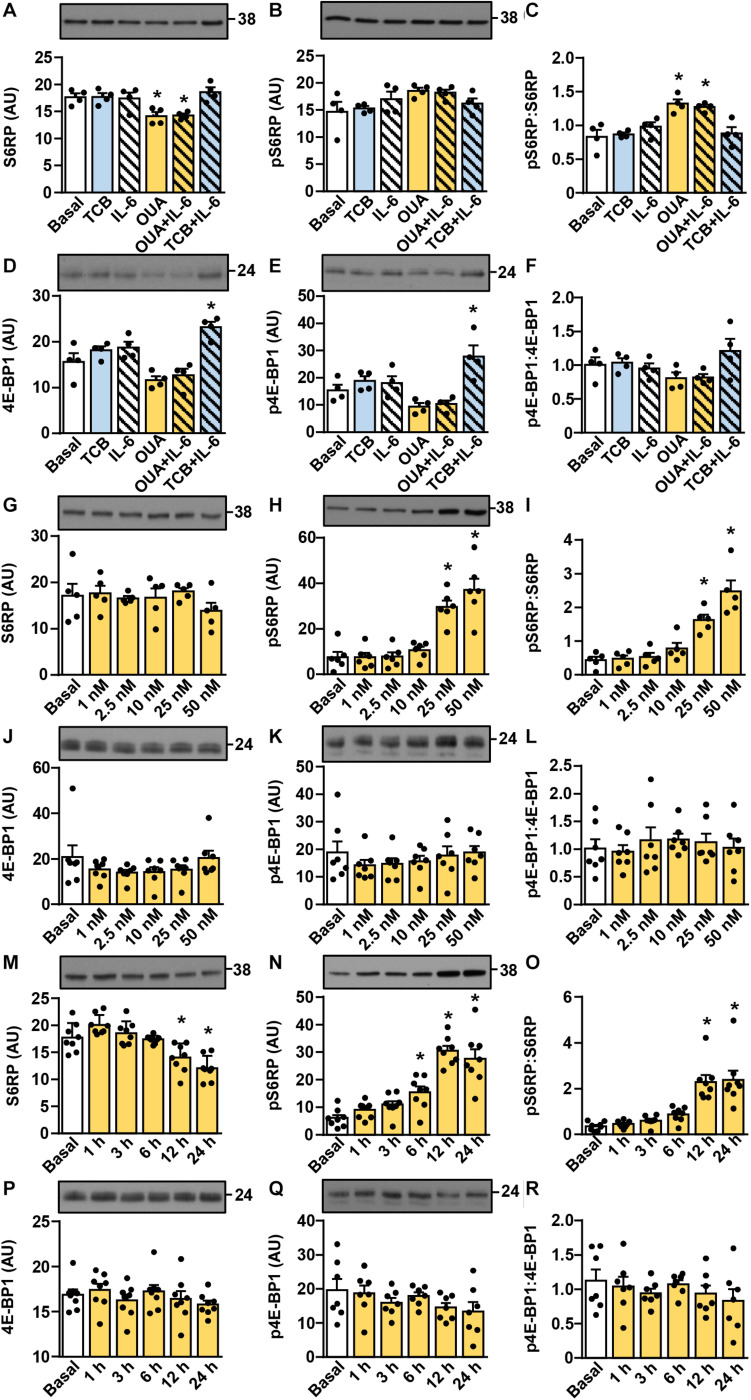
Ouabain stimulates the phosphorylation of S6RP in cultured human myotubes. **(A–F)** Human myotubes were treated with or without 50 nM ouabain (OUA) in Advanced MEM with 2% FBS for 20 h and in serum-free Advanced MEM for 4 h. Cells were treated with tocilizumab (100 μg/ml, TCB) during the last 4 h and with interleukin-6 (50 ng/ml, IL-6) during the last 15 min. **(G–R)** Human myotubes were treated in serum-free Advanced MEM with different concentrations of OUA for 24 h **(G–L)** or with 50 nM OUA for 1–24 h **(M–R)**. **(A,G,M)** The total S6RP, **(B,H,N)** phospho-S6RP (Ser235/236), **(D,J,P)** the total 4E-BP1, and **(E,K,Q)** phospho-4E-BP1 (Thr37/46) were measured by immunoblotting. Graphs in panels **(C,I,O)** show the phospho-S6RP:S6RP ratio and graphs in panels **(F,L,R)** show the phospho-4E-BP1:4E-BP1 ratio. Results are means with SEM (*n* = 4–8). *p < 0.05 vs. Basal.

Activation of the Akt-mTOR pathway (schematically presented in Figure 2M) is thought to link ouabain to cardiac hypertrophy (Liu et al., 2007). The mTOR pathway is activated by exercise and is important for hypertrophy of skeletal muscle (Baar and Esser, 1999; Terzis et al., 2008; Marabita et al., 2016). We assessed activity of this pathway (Figures 3, 4) by measuring the phosphorylation of Akt (Ser473), 70 kDa ribosomal protein S6 kinase (p70S6K, Thr389), S6 ribosomal protein (S6RP, Ser235/236), and the eukaryotic translation initiation factor 4E-binding protein 1 (4E-BP1, Thr37/46) (see Figure 2M). In cultured human myotubes the phosphorylation of Akt and/or the phospho-Akt:Akt ratios were increased by 10–50 nM ouabain (Figures 3A–C,E–G). An increase in the phosphorylation of Akt could be observed from 6 to 24 h of ouabain treatment (Figures 3I–K). Stimulation of the Akt phosphorylation was paralleled by an increase in the phosphorylation of p70S6K (Figures 3D,H,L). Phosphorylation of S6RP and/or the phospho-S6RP:S6RP ratio were also increased by ouabain (Figures 4A–C,G–I,M–O). Compared with Akt, higher concentrations of ouabain (25 or 50 nM) were required to stimulate the phosphorylation of p70S6K and S6RP. The phosphorylation of 4E-BP1 remained unaltered during treatment with ouabain (Figures 4D–F,J–L,P–R).

### Suppressive Effects of Ouabain on the Abundance of NKAα1 and STAT3 in Cultured Human Myotubes Are Mimicked by Puromycin and Blocked by MG-132

**FIGURE 5:**
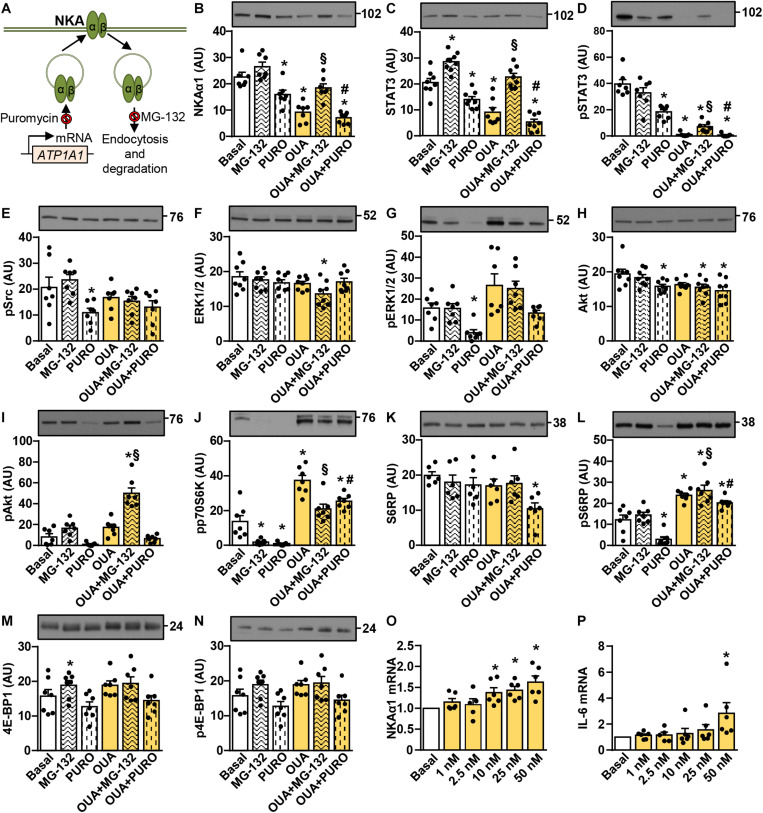
Suppressive effects of ouabain on the abundance of NKAα1 and STAT3 in cultured human myotubes are mimicked by puromycin and blocked by MG-132. **(A)** Puromycin and MG-132 inhibit mRNA translation and protein degradation, respectively. **(B–N)**: Human myotubes were treated in serum-free Advanced MEM with or without 1 μM MG-132 or 0.5 μg/ml puromycin (PURO) and/or 50 nM ouabain (OUA). MG-132 and puromycin were added 1 h before 24-h incubation with OUA. **(B)** α1-subunit of Na,K-ATPase (NKAα1), **(C)** the total STAT3, **(D)** phospho-STAT3 (Tyr705), **(E)** phospho-Src (Tyr527), **(F)** the total ERK1/2, **(G)** phospho-ERK1/2 (Thr202/Tyr204), **(H)** the total Akt, **(I)** phospho-Akt (Ser473), **(J)** phospho-p70S6K (Thr389), **(K)** the total S6RP, **(L)** phospho-S6RP (Ser235/236), **(M)** the total 4E-BP1 and **(N)** phospho-4E-BP1 (Thr37/46) were analyzed by immunoblotting. **(O,P)** Human myotubes were treated in serum-free Advanced MEM with different concentrations of OUA for 24 h. Expression of **(O)** NKAα1 mRNA and **(P)** interleukin-6 (IL-6) mRNA was determined with quantitative PCR. NKAα1 mRNA and IL-6 mRNA were normalized to three endogenous controls (PPIA mRNA, ACTB mRNA, and 18S rRNA). All values were normalized to respective Basal. Results are means with SEM (*n* = 6–8). *p < 0.05 vs. Basal, ^§^*p* < 0.05 vs. MG-132, ^#^*p* < 0.05 vs. puromycin.

Analysis of the Akt-mTOR pathway suggested that prolonged treatment with 25–50 nM ouabain stimulated protein synthesis (Figures 3, 4), while it suppressed the abundance of NKAα1 and STAT3. To investigate the mechanisms by which ouabain reduces the abundance of NKAα1 and STAT3, we pretreated the cells for 1 h with puromycin, an inhibitor of protein translation, and MG-132, an inhibitor of the proteasome (Figure 5A). After 1-h pretreatment with puromycin and MG-132, 50 nM ouabain was added for 24 h. Puromycin and ouabain reduced the abundance of NKAα1 and STAT3 (Figures 5B,C). The combined treatment did not suppress NKAα1 additively (Figure 5B), but it tended to further reduce the abundance of the total STAT3 (Figure 5C). The abundance of NKAα1 and STAT3 was higher in myotubes treated with MG-132 than in those treated with MG-132 and ouabain (Figures 5B,C). Ouabain stimulated dephosphorylation of STAT3 in the presence of MG-132 (Figure 5D), although MG-132 prevented ouabain-induced reduction of the total STAT3 (Figure 5C). This result again indicates that ouabain-induced dephosphorylation of STAT3 is not exclusively caused by a reduction of the total STAT3.

Ouabain tended to increase the phosphorylation of ERK1/2 in the presence of MG-132 and puromycin (Figures 5F,G), but it again had no effect on the phosphorylation of Src (Figure 5E). MG-132 markedly suppressed the basal phosphorylation of p70S6K (Figure 5J), consistent with its inhibitory effect on this kinase (Liu et al., 2011). Despite the suppression of the basal phosphorylation of p70S6K, MG-132 did not prevent the ouabain-stimulated activation of the Akt-mTOR pathway as assessed by measuring the phosphorylation of Akt (Figures 5H,I), p70S6K (Figure 5J), or S6RP (Figures 5K,L). While puromycin reduced the basal and the ouabain-stimulated phosphorylation of Akt (Figures 5H,I), p70S6K (Figure 5J) and S6RP (Figures 5K,L) cells remained responsive to ouabain. None of the treatments had a marked effect on 4E-BP1 (Figures 5M,N).

Taken together, these results suggested that ouabain reduced the abundance of NKAα1 and STAT3 by reducing protein synthesis and/or stimulating proteolysis. On the other hand, activation of the mTOR pathway would tend to stimulate protein synthesis and we therefore hypothesized that downregulation of these proteins might be due to reduced gene expression. However, ouabain upregulated NKAα1 mRNA (Figure 5O), demonstrating that the abundance of NKAα1 protein was not reduced due to the suppression of NKAα1 (*ATP1A1*) gene expression. Finally, since STAT3 is known to promote the *IL-6* gene expression in cultured rat myotubes (Bustamante et al., 2014), we evaluated if the ouabain-induced suppression of STAT3 signaling downregulates IL-6 expression. Despite the suppression of STAT3 signaling, ouabain increased IL-6 mRNA in a concentration-dependent manner (Figure 5P), again showing that the ouabain treatment did not lead to overall suppression of transcription.

### Ouabain Promotes the Cytokine Secretion From the Cultured Human Myotubes

**FIGURE 6:**
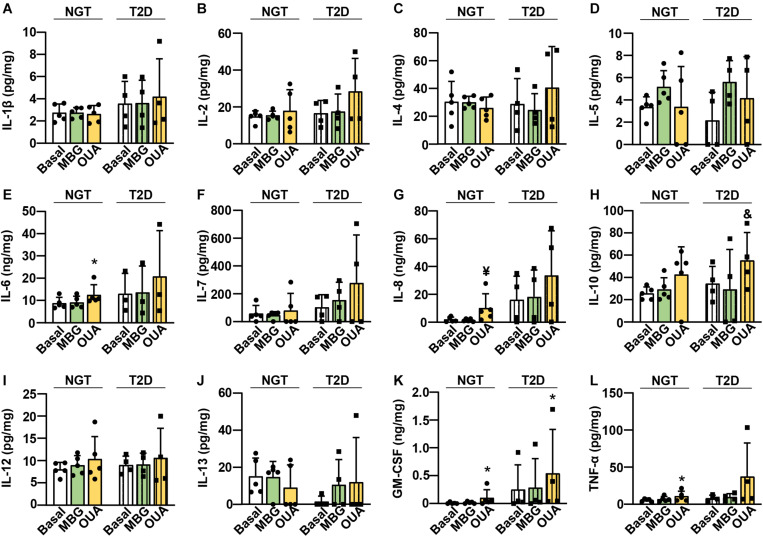
Ouabain promotes the cytokine secretion from the cultured human myotubes. Myotubes from subjects with normal glucose tolerance (NGT) or the type 2 diabetes (T2D) were treated with 100 nM ouabain (OUA) or 10 nM marinobufagenin (MBG) for 16 h. **(A–L)** Cytokines IL-1β, IL-2, IL-4, IL-5, IL-6, IL-7, IL-8, IL-10, IL-12, IL-13, GM-CSF, TNF-α were measured using High Sensitivity Human Cytokine Multiplex Immunoassay. The amount of secreted cytokines (in pg or ng) was normalized to the total cellular protein content (in mg). Results are means with SD (*n* = 3–5). *p < 0.05, ^¥^*p* = 0.054, ^&^*p* = 0.068 vs. Basal.

The ouabain-induced increase in IL-6 mRNA (Figure 5P) indicated that ouabain may increase the secretion of IL-6. To determine whether CTS modulate cytokine secretion from human myotubes, we used ouabain as well as marinobufagenin. We treated myotubes from subjects with normal glucose tolerance (NGT) or the type 2 diabetes (T2D) (Table 1) with 100 nM ouabain or 10 nM marinobufagenin for 16 h in serum-free DMEM. We used the NGT and T2D myotubes because chronic inflammation and dysregulated cytokine action is known to contribute to metabolic derangements in diabetes (Donath and Shoelson, 2011), which could lead to different responses to CTS. We measured 13 different cytokines, among which only interferon-γ could not be consistently detected in cell culture medium (Figure 6). The NGT and T2D myotubes most robustly secreted IL-6 (Figure 6E), IL-8 (Figure 6G), and GM-CSF (Figure 6K). Consistent with an upregulation of IL-6 mRNA (Figure 5P), ouabain promoted the secretion of IL-6 from the NGT myotubes (Figure 6E). Ouabain also promoted the secretion of IL-8 (Figure 6G), GM-CSF (Figure 6K), and TNF-α (Figure 6L). In the T2D myotubes, ouabain promoted the secretion of IL-10 (Figure 6H) and GM-CSF (Figure 6K). Marinobufagenin did not significantly alter the cytokine secretion, although IL-5 (Figure 6D) tended to be increased upon marinobufagenin treatment.

### HIF-1 Does Not Mediate the Ouabain-Induced Suppression of the IL-6/STAT3 Signaling in Cultured Human Skeletal Muscle Cells

**FIGURE 7:**
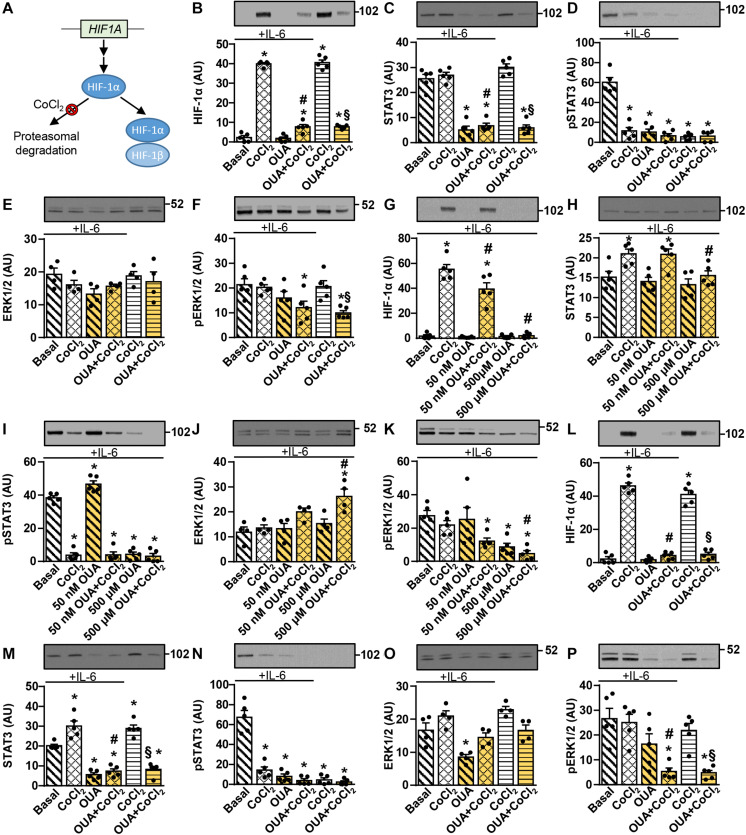
Ouabain blocks CoCl_2_-induced upregulation of HIF-1α in cultured human myotubes and myoblasts. **(A)** Under normoxic conditions HIF-1α is continuously hydroxylated (not shown) and degraded in the proteasome. CoCl_2_ blocks HIF-1α hydroxylation, thus suppressing its proteasomal degradation, which leads to heterodimerization with HIF-1β. **(B–F)** Human myotubes were incubated in Advanced MEM with 2% FBS with or without 50 nM ouabain (OUA) for 20 h. During the next 4 h cells were incubated in serum-free Advanced MEM with or without 50 nM OUA and/or 250 μM CoCl_2_. During the final 15 min, cells were treated with or without 50 ng/ml interleukin-6 (IL-6). **(G–K)** Human myoblasts were incubated in serum-free Advanced MEM with or without 50 nM or 500 μM OUA and/or 250 μM CoCl_2_ for 4 h. Cells were treated with 50 ng/ml IL-6 during the final 15 min. **(L–P)** Human myoblasts were incubated in Advanced MEM with 10% FBS with or without 50 nM OUA for 20 h. During the next 4 h cells were incubated in serum-free Advanced MEM with or without 50 nM OUA and/or 250 μM CoCl_2_. During the final 15 min, cells were treated with or without 50 ng/ml IL-6. **(B,G,L)** HIF-1α, **(C,H,M)** the total STAT3, **(D,I,N)** phospho-STAT3 (Tyr705), **(E,J,O)** the total ERK1/2 and **(F,K,P)** phospho-ERK1/2 were measured by immunoblotting. Results are means with SEM (*n* = 4–5). *p < 0.05 vs. Basal; ^#^*p* < 0.05 vs. CoCl_2_ + IL-6; ^§^*p* < 0.05 vs. CoCl_2_.

The phosphorylation of STAT3 was suppressed by 2.5 nM ouabain (Figures 1F–H), which did not alter activity of the ERK1/2 (Figures 2F–H) or the Akt-mTOR pathways (Figures 3E–H, 4G–L), suggesting that other pathways might be involved in suppressing the STAT3 signaling. Gene silencing of HIF-1α, the oxygen-sensitive subunit of the heterodimeric transcription factor HIF-1 (HIF-1α/HIF-1β) (Samanta and Semenza, 2017), suppresses the phosphorylation of STAT3 in fibroblasts (Gao et al., 2015), while ouabain reduces HIF-1α levels in cancer and smooth muscle cells (Zhang et al., 2008; Koltsova et al., 2014). Based on these reports we hypothesized that ouabain may block the IL-6-stimulated phosphorylation of STAT3 by suppressing HIF-1. To examine this possibility, we treated myotubes with 50 nM ouabain for 20 h in Advanced MEM supplemented with 2% FBS. This was followed by a 4-h treatment in serum-free Advanced MEM with 50 nM ouabain with or without 250 μM CoCl_2_, which prevents normoxic degradation of HIF-1α (Figure 7A) and was shown to effectively induce HIF-1α in human myoblasts (Pirkmajer et al., 2010; Lojk et al., 2015). Recombinant human IL-6 was added during the last 15 min of the experiment (Figures 7B–F). In the absence of ouabain, CoCl_2_ potently upregulated HIF-1α (Figure 7B). Ouabain markedly suppressed HIF-1α upregulation (Figure 7B) as well as the abundance of the total and phosphorylated STAT3 (Figures 7C,D). CoCl_2_ did not alter the total STAT3, but the phosphorylation of STAT3 was blocked by CoCl_2_ even in the presence of IL-6 (Figures 7C,D). Interestingly, ouabain suppressed the phosphorylation of ERK1/2 in the presence of CoCl_2_ (Figures 7E,F).

To further evaluate the role of HIF-1α, we wanted to perform gene silencing, which is more efficient in myoblasts than in myotubes. Before performing gene silencing, we evaluated how ouabain affects the HIF-1α and STAT3 signaling in myoblasts. Proliferating human myoblasts were treated with ouabain (50 nM or 500 μM) and CoCl_2_ (250 μM) for 4 h and IL-6 was added for the last 15 min (Figures 7G–K). Treatment with CoCl_2_ markedly upregulated HIF-1α levels (Figure 7G) and suppressed the phosphorylation of STAT3 (Figures 7H,I). In the presence of IL-6, 50 nM ouabain slightly suppressed HIF-1α and somewhat increased the phosphorylation of STAT3 (Figures 7G,I). In contrast, 500 μM ouabain, which is sufficient to completely inhibit NKA transport activity (Kotova et al., 2006a), markedly suppressed HIF-1α (Figure 7G) and the phospho-STAT3 levels (Figure 7I). All treatments reduced or tended to reduce the phosphorylation of ERK1/2 (Figures 7J,K).

We then pretreated myoblasts with 50 nM ouabain for 20 h in the presence of 10% FBS in Advanced MEM (Figures 7L–P), which was followed by a 4-h treatment with 50 nM ouabain and/or 250 μM CoCl_2_ in serum-free medium. IL-6 was added during the final 15 min. Pretreatment with 50 nM ouabain was sufficient to almost completely suppress HIF-1α (Figure 7L) and phospho-STAT3 (Figures 7M,N). The phosphorylation of ERK1/2 was again reduced by all treatments (Figures 7O,P). Taken together, these results replicated ouabain effects in myotubes, indicating myoblasts were a valid model to test whether HIF-1α mediates suppressive effects of ouabain on STAT3.

**FIGURE 8:**
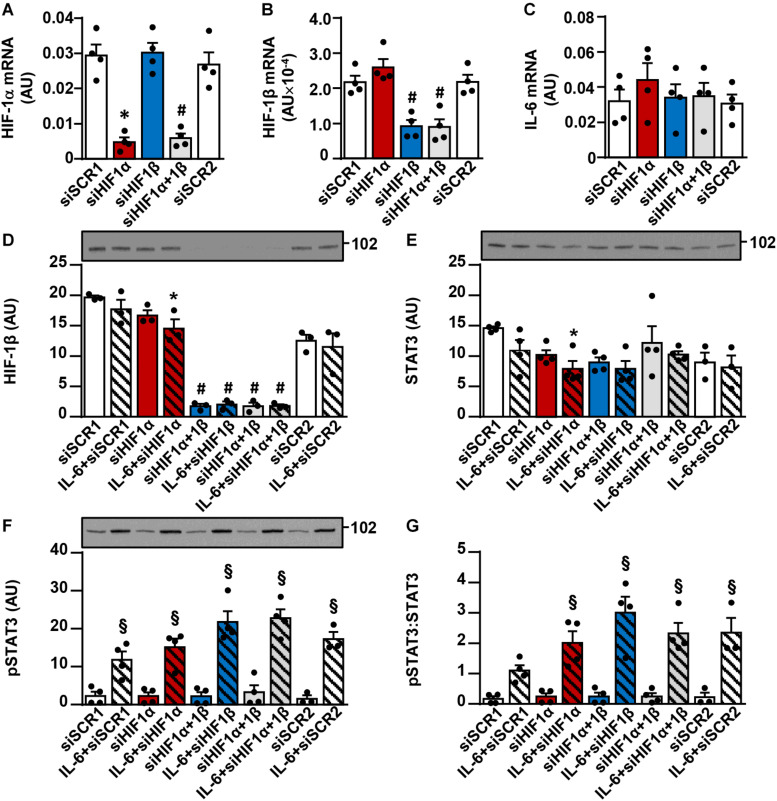
Gene silencing of HIF-1α and/or HIF-1β does not suppress the IL-6/STAT3 signaling in cultured human myoblasts. Human myoblasts were treated with siRNA directed against HIF-1α (siHIF1α) and/or HIF-1β (siHIF1β). 44 h after application of siRNA cells were serum-starved for 4 h and then analyzed for **(A)** HIF-1α mRNA, **(B)** HIF-1β mRNA, and **(C)** IL-6 mRNA by the quantitative PCR or **(D–G)** treated with 50 ng/ml interleukin-6 (IL-6) for 15 min and analyzed for **(D)** HIF-1β, **(E)** the total STAT3 and **(F)** phospho-STAT3 (Tyr705) by immunoblotting. Graph in panel **(G)** shows the phospho-STAT3:STAT3 ratio. Two concentrations of scrambled siRNA (siSCR) were used as controls: 5 nM (siSCR1; control for siHIF-1α) or 45 nM (siSCR2; control for siHIF-1β and siHIF-1α + 1β). mRNA of target genes is expressed relative to mRNA of a reference gene (ACTB). Results are means with SEM (*n* = 3–4). *p < 0.05 vs. siSCR1; ^#^*p* < 0.05 vs. siSCR2; ^§^*p* < 0.05 vs. respective basal.

To examine whether suppression of HIF-1α mimics effects of ouabain on IL-6 signaling, we knocked down HIF-1α and/or HIF-1β in human myoblasts (Figure 8) using a previously validated protocol, which effectively suppresses capacity of human myoblasts to activate the HIF-1α/HIF-1β pathway (Pirkmajer et al., 2010; Lojk et al., 2015). Treatment with HIF-1α and/or HIF-1β siRNA resulted in a marked reduction of HIF-1α mRNA (Figure 8A), HIF-1β mRNA (Figure 8B), and/or the abundance of HIF-1β protein (Figure 8D). In contrast, IL-6 mRNA was not reduced (Figure 8C), indicating HIF-1 (HIF-1α/HIF-1β) did not contribute to the expression of *IL-6* gene under the basal conditions. To test whether ouabain might suppress IL-6 signaling by downregulating HIF-1, we treated HIF-1α and/or HIF-1β deficient myoblasts with IL-6 for 15 min. As assessed by the phosphorylation of STAT3, gene silencing of HIF-1α and/or HIF-1β did not suppress the IL-6/STAT3 signaling (Figures 8E–G). Taken together, these results suggest that the suppression of HIF-1 does not mediate effects of ouabain on the IL-6/STAT3 signaling.

### Ouabain Does Not Suppress the Phosphorylation of STAT3 and the Induction of HIF-1α in Rat L6 Myotubes

**FIGURE 9:**
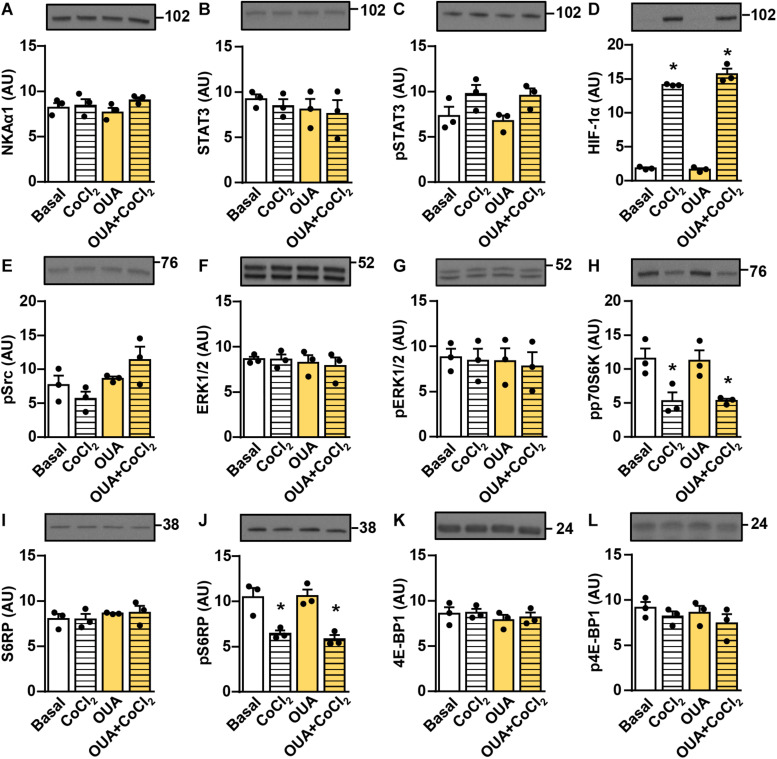
Ouabain does not suppress STAT3 and HIF-1α in rat L6 myotubes. L6 myotubes were treated in serum-free MEMα with or without 50 nM ouabain (OUA) for 24 h and with or without 250 μM CoCl_2_ during the last 4 h. **(A)** α1-subunit of Na,K-ATPase (NKAα1), **(B)** the total STAT3, **(C)** phospho-STAT3 (Tyr705), **(D)** HIF-1α, **(E)** phospho-Src (Tyr527), **(F)** the total ERK1/2, **(G)** phospho-ERK1/2 (Thr202/Tyr204), **(H)** phospho-p70S6K (Thr389), **(I)** the total S6RP, **(J)** phospho-S6RP (Ser235/236), **(K)** the total 4E-BP1 and **(L)** phospho-4E-BP1 (Thr37/46) were measured by immunoblotting. Results are means with SEM (*n* = 3). *p < 0.05 vs. Basal.

The α-subunit of NKA is a physiological receptor for ouabain and related CTS, but other receptors may also play a role (Askari, 2019b). Unlike human myotubes, which prominently express both NKAα1 and NKAα2 (the NKAα1:NKAα2 mRNA ratio ≈ 2:1), rat L6 myotubes express almost exclusively NKAα1 (the NKAα1:NKAα2 mRNA ratio ≈ 100:1) (Table 2). The rodent NKAα1 is ouabain-resistant (Akera et al., 1969; Fallows et al., 1987; Kent et al., 1987; Price and Lingrel, 1988) and its transport activity is not reduced even by 1 μM ouabain (Chibalin et al., 2012). To evaluate whether the suppression of STAT3 signaling and HIF-1α is dependent on NKAα1, we treated the rat L6 myotubes with 50 nM ouabain and/or CoCl_2_. The abundance of NKAα1 (Figure 9A) as well as the abundance and the phosphorylation of STAT3 (Figures 9B,C) were unaltered by the ouabain treatment. CoCl_2_ markedly upregulated HIF-1α, but in contrast to the human skeletal muscle cells (Figure 7) ouabain did not affect the abundance of HIF-1α in CoCl_2_-treated cells (Figure 9D). Ouabain did not have any major effect on Src, ERK1/2, or mTOR signaling (Figures 9E–L). Taken together, these results indirectly suggest that ouabain requires the ouabain-sensitive NKAα1 to modulate the ERK1/2 and the mTOR pathways and to suppress the STAT3 and the HIF-1α signaling in skeletal muscle cells.

**TABLE 2:** Gene expression of the NKAα isoforms in cultured human and rat L6 myotubes.

NKAα isoform	Human myotubes (×10^–2^)	Rat L6 myotubes (×10^–3^)
NKAα1	12.8 ± 5.5	1.84 ± 0.19
NKAα2	6.27 ± 4.05	0.018 ± 0.001***
NKAα3	0.027 ± 0.043**	Not detected

Expression levels are reported as the gene expression ratios (NKAα mRNA/actin-β mRNA). Results are shown as means ± SD (human myotubes: *n* = 6, rat L6 myotubes: *n* = 4). **p < 0.01 vs. NKAα1 in human myotubes; ***p < 0.0001 vs. NKAα1 in rat L6 myotubes.

## Discussion

Cardiotonic steroids, especially ouabain, are thought to be secreted during exercise in dogs and humans (Valdes et al., 1988; Antolovic et al., 2000; Bauer et al., 2005). Skeletal muscles, which contain a major pool of CTS receptor NKA, are an important target tissue for the exogenous ouabain (Kjeldsen et al., 1985; Clausen, 1996, 2010). If NKA is a hormonal receptor for the endogenous CTS (Schoner, 2002; Dostanic-Larson et al., 2005; Bagrov et al., 2009; Aperia et al., 2016; Blaustein, 2018), skeletal muscles would be an important site of action of the endogenous ouabain or the related CTS during exercise. Using cultured human skeletal muscle cells we found that ouabain suppressed the IL-6/STAT3 signaling and promoted the secretion of IL-6 and several other cytokines. These results implicate a role for NKA and CTS in regulation of the IL-6 signaling and secretion in skeletal muscle. In addition, ouabain suppressed HIF-1α and activated the ERK1/2 and the mTOR pathways, which are all involved in signaling response to muscle contractions and exercise (Egan and Zierath, 2013). Taken together, our results imply that the putative endogenous ouabain or CTS that are still used for therapy of certain types of heart disease could modulate muscle adaptations to exercise.

**FIGURE 10:**
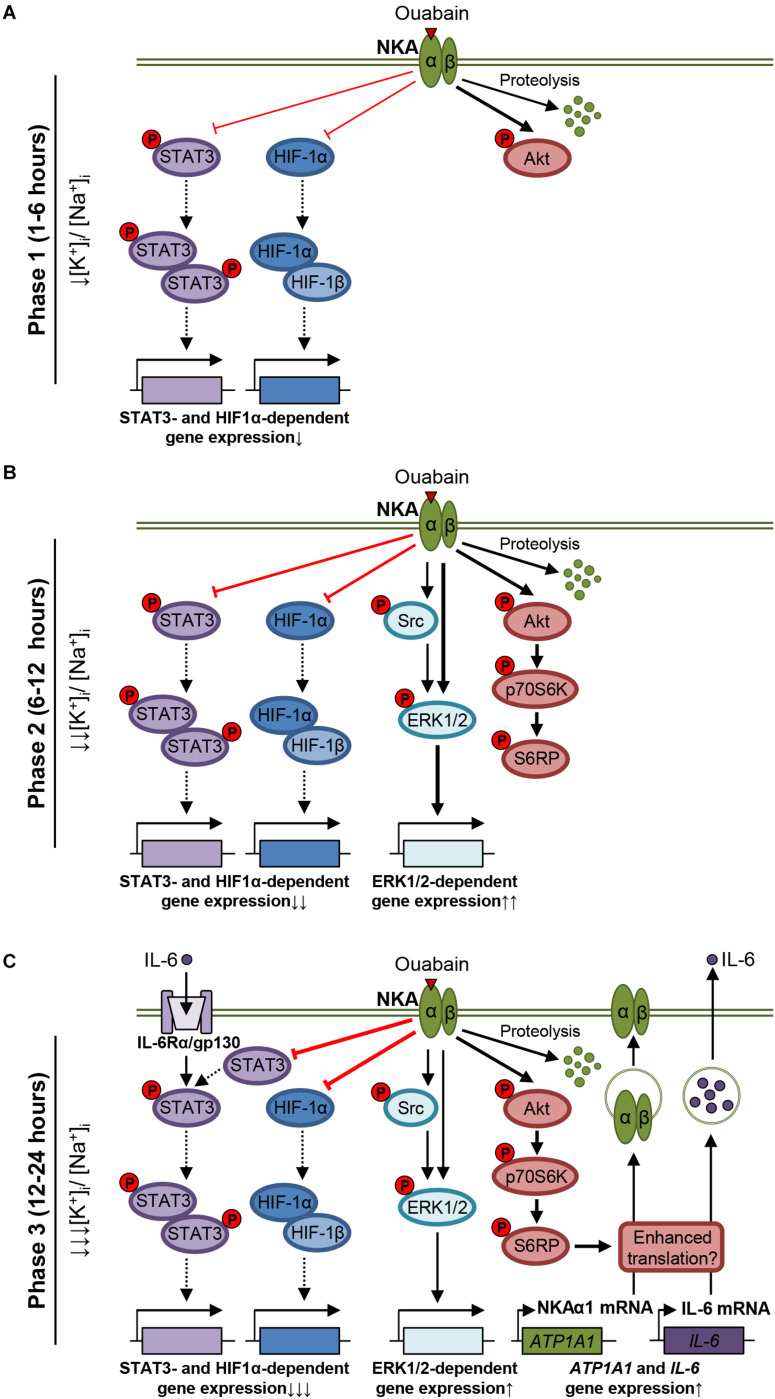
Overview of ouabain effects in cultured human skeletal muscle cells. **(A)** In the first phase (1–6 h), ouabain stimulates proteolysis of NKA and starts to suppress the basal phosphorylation of STAT3 and the HIF-1 pathway. The Akt pathway is activated. **(B)** In the second phase (6–12 h), ouabain activates the ERK1/2 pathway, possibly via Src-dependent and independent mechanisms, as well as the mTOR pathway. **(C)** In the third phase (12–24 h), ouabain upregulates expression of NKAα1 (*ATP1A1* gene) as well as expression and secretion of IL-6. The IL-6/STAT3 and the HIF-1 pathways are markedly suppressed (Note: Alterations in the [K^+^]_i_:[Na^+^]_i_ ratio are inferred based on the published data. The thickness of lines indicates the intensity of the effects).

STAT3, activated in skeletal muscle by IL-6 and exercise, plays a role in skeletal muscle metabolism, regeneration, and hypertrophy (Trenerry et al., 2007; Pedersen and Febbraio, 2008; Serrano et al., 2008; Yin et al., 2013). Ouabain suppressed signaling via STAT3 under the basal and the IL-6-stimulated conditions (Figures 1, 7). The suppression of the STAT3 pathway was likely the result of two different mechanisms. First, the abundance of the total STAT3 was reduced by ouabain, which in itself reduces the amount of STAT3 that is available for phosphorylation. Consistent with our observation, ouabain reduced the abundance of STAT3 in the alveolar epithelial cells by inhibiting translation (Amarelle et al., 2019). Second, ouabain likely suppressed the phosphorylation of STAT3 independently of alterations in the abundance of STAT3. This idea is supported by the observation that the dephosphorylation of STAT3 preceded the loss of the total STAT3 in our time-course experiment (Figures 1J–L). Further, lower ouabain concentrations (2.5–25 nM) were needed to reduce the phosphorylation of STAT3 than its abundance (50 nM) (Figures 1F–H). Finally, ouabain markedly suppressed the phosphorylation of STAT3 in cells treated with MG-132 although the total STAT3 was unaltered (Figures 5C,D). In sum, our data indicate that ouabain is a potent suppressor of the IL-6/STAT3 signaling (Figure 10).

As well as STAT3, ouabain markedly reduced the abundance of NKAα1 in human myotubes (Figures 1A,E,I). NKAα1 can be modified by ubiquitination (Coppi and Guidotti, 1997) and MG-132 opposes the loss of NKAα1, which is caused by various treatments, including CdCl_2_ (Thevenod and Friedmann, 1999) and hypoxia (Comellas et al., 2006). Actions of MG-132 could indicate that NKAα1 is ubiquitinated and at least partially degraded in the proteasome in response to the ouabain treatment. However, the ubiquitination of NKAα1 does not necessarily mean that it is directed to the proteasome. Indeed, ubiquitination might be more important as regulator of its endocytosis and lysosomal degradation (Lecuona et al., 2009). Inhibition of the proteasome by MG-132 reduces the availability of ubiquitin (Melikova et al., 2006), which may in turn reduce the ubiquitination of NKAα1, thus increasing its retention in the membrane. Taken altogether, our results indirectly suggest that ouabain induces NKAα1 ubiquitination, which subsequently leads to its internalization and proteolysis (Figure 10) in the lysosome and/or proteasome.

Prolonged exposure to ouabain upregulated NKAα1 mRNA (Figure 5O) and stimulated the phosphorylation of Akt, p70S6K, and S6RP (Figures 3, 4), indicating activation of the Akt-mTOR pathway, which would tend to stimulate the protein synthesis. Activation of the ERK1/2 pathway (Figure 2) might have also contributed to the activation of the mTOR pathway (Figure 2M). However, activation of the Akt-mTOR pathway does not exclude the possibility that inhibition of translation contributed to the loss of NKAα1. For instance, inhibition of NKA might have reduced the [K^+^]_i_:[Na^+^]_i_ ratio, thus suppressing translation via a mechanism that does not directly involve protein kinases. Indeed, in the epithelial alveolar cells ouabain inhibits NKA, decreases [K^+^]_i_, and thereby reduces protein translation that leads to the loss of STAT3 (Amarelle et al., 2019). The reduced abundance of STAT3 in skeletal muscle cells treated with 50 nM ouabain for 24 h indirectly suggests a similar mechanism exists in skeletal muscle. Activation of the mTOR pathway during the postexercise recovery is linked to protein synthesis and muscle hypertrophy (Baar and Esser, 1999; Terzis et al., 2008). Considered together, our data imply that the endogenous or the exogenous ouabain might modulate response of the mTOR pathway (Figure 10) and translational machinery in skeletal muscle after exercise.

The EGFR/Src/ERK1/2 pathway established NKA as a signal transducer (Xie and Askari, 2002), but its functional significance is still debated (Askari, 2019a). ERK1/2 is activated by exercise and is thought to be an important transcriptional regulator in skeletal muscle (Widegren et al., 1998; Yu et al., 2003). While we did not study this pathway in detail, our results suggest that lower concentrations of ouabain (1–25 nM) do not potently stimulate the phosphorylation of ERK1/2 (Figures 2F–H). Indeed, even 50 nM ouabain increased its phosphorylation only with the prolonged treatment (12–24 h). Alternatively, the signal might be transient and returns to basal within the first hour of ouabain treatment. A long delay in ERK1/2 response indirectly suggests that it might be due to altered [K^+^]_i_, [Na^+^]_i_, or [Ca^2+^]_i_ rather than a direct result of activation of the EGFR/NKA/Src signaling complex. However, a more rapid ERK1/2 response can be observed in myotubes treated with 100 nM ouabain or 10 nM marinobufagenin (Kotova et al., 2006a), which is consistent with the idea that ERK1/2 activation is strongly dependent on the concentration of ouabain (Wu et al., 2015). While our data suggest that low concentrations of ouabain stimulate ERK1/2 only with a significant delay, this does not mean that such activation would not be physiologically important. For instance, a role of the putative endogenous ouabain could be to stimulate ERK1/2 and the glycogen synthesis (Kotova et al., 2006a, b) during the postexercise recovery.

Ouabain suppressed the CoCl_2_-induced expression of HIF-1α in human skeletal muscle cells in a time- and concentration-dependent manner (Figure 7), consistent with suppressive effect of ouabain and other CTS on the HIF-1α abundance in cancer (Zhang et al., 2008) and smooth muscle cells (Koltsova et al., 2014). In contrast, some studies suggested that ouabain or digoxin may upregulate HIF-1α in colon cancer cells and the kidney tubules (Rosenberger et al., 2006; Riganti et al., 2009). Differences in ouabain effects could be due to different experimental models or ouabain concentrations. For instance, colon cancer cells were treated with 1 μM ouabain or digoxin (Riganti et al., 2009). In cultured human skeletal muscle cells under oxygen-deficient conditions or CoCl_2_ treatment, HIF-1α is rapidly upregulated and stimulates expression of hypoxia-responsive genes (Pirkmajer et al., 2010; Lojk et al., 2015). HIF-1 is also upregulated in contracting skeletal muscles and plays a role in adaptations to exercise (Ameln et al., 2005; Egan and Zierath, 2013). Taken together with studies demonstrating ouabain secretion during exercise (Antolovic et al., 2000; Bauer et al., 2005), our results suggest ouabain may modulate the hypoxia-induced gene expression (Figure 10) in skeletal muscle.

Ouabain decreased the responsiveness of human myotubes to IL-6 (Figures 1, 7), but promoted the secretion of IL-6 (Figure 6). This dual effect suggests ouabain might be involved in a negative feedback regulation of IL-6 in skeletal muscle, whereby an initial decrease in responsiveness results in increased IL-6 secretion, which would tend to oppose the suppression of the IL-6/STAT3 pathway. IL-6 plays a pro-inflammatory role in the stress response during acute illness and chronic inflammation, such as rheumatoid arthritis (Friedland et al., 1992; Smolen et al., 2008; Febbraio et al., 2010; Hocevar et al., 2013; Pal et al., 2014). In critical illness, plasma concentrations of the endogenous ouabain are increased and correlate with high concentrations of IL-6 (Berendes et al., 2003). Chronic exposure to the increased IL-6 concentrations has been linked to deleterious effects, such as loss of skeletal muscle mass (Tsujinaka et al., 1995, 1996; Haddad et al., 2005; Bodell et al., 2009) and insulin resistance (Glund and Krook, 2008; Naugler and Karin, 2008). During the stress response, secretion of anti-inflammatory and immunosuppressive adrenocortical hormones, especially cortisol, is essential to keep inflammation and activation of immune system in check (Russell and Lightman, 2019). By blocking the IL-6 action, ouabain may dampen negative effects of exposure to high IL-6 concentrations in skeletal muscle and other tissues. For instance, ouabain could protect skeletal muscle from overstimulation with IL-6 during contractions when the concentrations of IL-6 in skeletal muscle and the peritendinous tissue can be as high as ∼2000–3000 pg/ml or more (Langberg et al., 2002; Rosendal et al., 2005). Notably, these concentrations are already in the range of plasma concentrations observed during severe sepsis (Friedland et al., 1992).

The ouabain-induced cytokine expression and secretion are probably not exclusively regulated by inhibition of the transport activity of NKA. If they were, ouabain and marinobufagenin would have a similar effect on the cytokine secretion. Importantly, we used 100 nM ouabain and 10 nM marinobufagenin, which were previously shown to inhibit NKA in cultured myotubes to a similar degree (Kotova et al., 2006a). Nevertheless, inhibition of NKA likely plays at least a modulatory role since signaling pathways, transcriptional activity, and protein translation are sensitive to a decrease in [K^+^]_i_ and/or an increase in [Na^+^]_i_ (Kuroki et al., 1997; Klimanova et al., 2017, 2019; Orlov et al., 2017; Amarelle et al., 2019). Effects of CTS on the cytokine expression or secretion seem to depend on many factors, including cell type, the timing, and CTS concentration. For instance, ouabain and marinobufagenin increased expression of IL-6 mRNA and/or the IL-6 secretion in fibroblasts and cytotrophoblast cells (Akashi et al., 1990; Uddin et al., 2008). In contrast, ouabain increased the TNF-α secretion, while reducing the IL-6 secretion in mononuclear cells (Foey et al., 1997). Similarly, digoxin blocked the lipopolysaccharide-stimulated secretion of IL-6, IL-8, and TNF-α from mononuclear cells (Ihenetu et al., 2008), but promoted the expression of TNF-α and IL-6 in the heart of mice with myocarditis (Matsumori et al., 1999). Finally, it has to be considered that some effects of CTS on the cytokine secretion might be indirect. For instance, here we showed that ouabain promotes the secretion of TNF-α, which is known to stimulate the IL-6 secretion from the human skeletal muscle cells (Prelovsek et al., 2006).

One caveat regarding the physiological relevance of our results are the ouabain (1–100 nM) and marinobufagenin (10 nM) concentrations that we used for treatment of cells. Some reports suggest that the plasma concentrations of the endogenous CTS could be in the range of ∼0.5–2 nM, although others indicated CTS concentrations could be much lower (Gottlieb et al., 1992; Manunta et al., 1999, 2006; Berendes et al., 2003; Bagrov et al., 2009; Pavlovic, 2020). On the other hand, the ouabain plasma concentrations of up to 15–20 nM were measured in some subjects receiving high-salt diet (Manunta et al., 2006) and concentrations of ouabain or ouabain-like substance may perhaps transiently reach 80–170 nM during exercise (Bauer et al., 2005). While steady-state plasma concentrations of the exogenous ouabain are in the high picomolar range (close to 1 nM), intravenous administration of ouabain results in peak plasma concentrations as high as 5–16 nM depending on the dose (Selden and Smith, 1972; Lewis et al., 1994; Pidgeon et al., 1994), indicating that it is not incompatible with survival if the endogenous ouabain or ouabain-like substance would reach 15–20 nM in plasma. However, whether concentrations of the endogenous ouabain as high as 100 nM or more are possible has been debated, not least because the specificity of the ouabain immunoassay has been questioned (Forni, 2005; Hilton and McKinnon, 2005).

In our study ouabain had most pronounced effects during the prolonged treatments (6–24 h), while signaling pathways that we analyzed did not respond during the first 3 h of treatment. In contrast, ouabain immunoreactivity rapidly declined (the half-time was in the order of minutes) once exercise bout had been completed (Bauer et al., 2005), which raises questions whether late effects of ouabain in the cell culture are physiologically relevant. The pharmacokinetic studies demonstrated that ouabain displays a biphasic decline (Selden and Smith, 1972; Lewis et al., 1994; Pidgeon et al., 1994). In the first phase elimination is rapid, while the second (late) phase displays slow elimination with half-times as long as 20 h and more (Selden and Smith, 1972). After intravenous application in humans, the plasma ouabain concentrations during the late phase of elimination are in the 1–2 nM range. We showed that 2.5 nM ouabain is sufficient to suppress the abundance of NKAα1 (Figure 1E) and the phosphorylation of STAT3 (Figure 1G) after 24-h treatment. Considered together with the pharmacokinetic data (Selden and Smith, 1972; Lewis et al., 1994; Pidgeon et al., 1994), our results indirectly suggests that ouabain may modulate the IL-6/STAT3 signaling *in vivo*. However, based on the kinetics of ouabain-induced responses in the cell culture, the putative endogenous ouabain probably would not act as a rapid regulator of the cytokine secretion and action, but rather as a modulator that exerts its biological effects for hours after completion of exercise.

Physiological relevance of our results also needs to be discussed in the context of NKAα isoforms. In rat skeletal muscle NKAα2 is the predominant isoform (∼60–90%), although NKAα1 is also prominently expressed (∼10–40%) (Orlowski and Lingrel, 1988; Sweadner et al., 1992; Hansen, 2001; He et al., 2001; Kristensen and Juel, 2010). Gene expression data suggest a similar situation in human skeletal muscle (Perry et al., 2013). Cultured human skeletal muscle cells express NKAα1 and NKAα2 (Al-Khalili et al., 2004; Murphy et al., 2004). Indeed, unlike cultured primary rat skeletal muscle cells, in which NKAα2 protein is below the detection limit (Sharabani-Yosef et al., 1999), human cells upregulate NKAα2 during the differentiation into myotubes (Al-Khalili et al., 2004). Although NKAα1 relatively predominates in human myotubes (Table 2) it has to be considered that human NKAα1 and NKAα2 are similarly sensitive to ouabain (Wang et al., 2001), suggesting that inhibition of both subunits likely contributed to cellular responses in our model. Whether ouabain also reduces the abundance of NKAα2, which predominates in skeletal muscle tissue, still needs to be examined. However, if marked downregulation of NKAα1 in the ouabain-treated myotubes is considered together with the functional differences between NKAα1, which is thought to be important for ion transport in resting skeletal muscle, and NKAα2, which likely has a major role in ion transport during contractions (Radzyukevich et al., 2004, 2013; DiFranco et al., 2015), our data suggest that ouabain might primarily modulate skeletal muscle function at rest. This would be consistent with the delayed signaling responses that we observed in this study as well as the idea that the putative endogenous ouabain might modulate muscle function during the postexercise recovery rather than during exercise.

Another important aspect is the lack of ouabain effect in the rat L6 cells (Figure 9). The L6 cells express almost exclusively the NKAα1 isoform, which is ouabain-resistant in rodents (Akera et al., 1969; Fallows et al., 1987; Kent et al., 1987; Price and Lingrel, 1988). Here we used the L6 cells to test whether a ouabain-sensitive NKAα isoform was required for suppression of IL-6/STAT3 signaling in order to exclude the possibility of non-specific actions via NKA-independent pathways. However, ouabain can modulate ion transport in rat skeletal muscle by inhibiting the ouabain-sensitive NKAα2 subunit (Chibalin et al., 2012). Further, low concentrations of ouabain (10–20 nM) were shown to affect the skeletal muscle function in rats (Kravtsova et al., 2020). Our results in L6 cells therefore do not exclude the possibility that ouabain modulates IL-6/STAT3 signaling in rat skeletal muscle.

## Conclusion

In conclusion, we showed that ouabain suppressed the IL-6/STAT3 signaling but promoted the secretion of IL-6 and other cytokines in cultured human skeletal muscle cells. Ouabain also modulated the ERK1/2, the mTOR, as well as the HIF-1α pathway, which are all involved in skeletal muscle responses to exercise (Egan and Zierath, 2013). However, it needs to be stressed that we observed no major signaling responses at least up to 3 h of treatment of ouabain. While we did not examine how skeletal muscle cells respond within the first hour, when rapid responses might have been observed, the delay indirectly suggests that alterations in cell signaling might be primarily due to altered [K^+^]_i_ and [Na^+^]_i_, caused by inhibition of NKA. Indeed, even the dephosphorylation of STAT3 required several hours to appear, possibly due to altered levels or activity of phosphatases or other proteins involved in its regulation.

The time course of ouabain effects on the abundance of NKAα1 and STAT3, combined with the increased IL-6 secretion, and expression of NKAα1 and IL-6 mRNA, indirectly suggest that human skeletal muscle cells go through several phases in response to ouabain (Figure 10). Our hypothesis is that in the first phase (1–6 h) (Figure 10A), NKAα1 is lost due to increase in its endocytosis and proteolysis. Inhibition of NKA, combined with the reduced abundance of NKA in the sarcolemma, leads to a progressive decrease in the [K^+^]_i_:[Na^+^]_i_ ratio, which suppresses translation and triggers transcriptional responses. This phase is also characterized by the STAT3 inactivation (dephosphorylation) and the Akt activation (phosphorylation), which appear by 6 h of ouabain treatment. In addition, based on results in myoblasts (Figure 7G), ouabain starts to suppress the HIF-1 pathway.

The second phase (6–12 h) (Figure 10B) is characterized by a continued and more pronounced loss of NKAα1 and further reductions in the [K^+^]_i_:[Na^+^]_i_ ratio. Toward the end of this phase, cells respond also with activation of the ERK1/2 and mTOR pathways.

In the third phase (12–24 h) (Figure 10C), the loss of STAT3, which has a relatively short half-life (∼4–8 h) (Siewert et al., 1999), suggests repression of translation (Amarelle et al., 2019). Ouabain markedly suppressed upregulation of HIF-1α by CoCl_2_ (Figure 7), which inhibits its proteolysis, which also suggests that translation is repressed after the 24-h treatment with ouabain. However, upregulation of transcription of the *ATP1A1* and *IL-6* genes, combined with activation of the mTOR pathway, and an increase in the IL-6 secretion indicates that the synthesis of specific proteins is increased, possibly to counteract the imbalance in a negative feedback manner (Figure 10C). Importantly, while the [K^+^]_i_:[Na^+^]_i_ ratio is most likely at its lowest level toward the end of the third phase (Lamb and McCall, 1972; Klimanova et al., 2017), the expression of IL-6 was shown to be upregulated when the [K^+^]_i_:[Na^+^]_i_ ratio is decreased in different types of cells (Koltsova et al., 2012), which is consistent with the increased IL-6 expression and secretion that we observed in myotubes.

In summary, our data are therefore compatible with the idea that inhibition of the NKA transport activity is an important mechanism of CTS action as highlighted by Orlov et al. (2017); Askari (2019a), and Klimanova et al. (2019). However, there are several important open questions in relation to the CTS action on cytokines in skeletal muscle that need to be addressed by future studies. First, mechanisms that link ouabain to dephosphorylation of STAT3 and delayed activation of the ERK1/2 and the Akt-mTOR pathways need to be identified. It would be particularly important to dissect the contribution of ionic vs. non-ionic mechanisms. Second, mechanisms underlying the selective regulation of the cytokine secretion by CTS should be established. Third, it also needs to be established whether the suppression of the IL-6/STAT3 signaling by ouabain is physiologically important. Finally, it would be relevant to examine if the suppression of the IL-6/STAT3 signaling by ouabain could be exploited therapeutically in inflammatory conditions, in which the IL-6/STAT3 pathway is pathologically activated.

## Data Availability Statement

The raw data supporting the conclusions of this article will be made available by the authors, without undue reservation.

## Ethics Statement

The studies involving human participants and preparation of primary human skeletal muscle cells and experimental procedures involving these cells were reviewed and approved by the Republic of Slovenia National Medical Ethics Committee (ethical approval no. 71/05/12 and 0120-698/2017/4) or Ethics Committee at Karolinska Institutet (ethical approval no. DNR 2006/225-31/1). The patients/participants provided their written informed consent to participate in this study.

## Author Contributions

SP and AC conceptually designed the study. SP, AC, LK, TM, and KP planned the experiments. SP wrote the first draft of the manuscript. SP, KB, UM, KaM, KsM, KD, KG, and LJ did the experiments. All authors participated in data analysis and writing of the manuscript. SP and AC provided the funding.

## Conflict of Interest

The authors declare that the research was conducted in the absence of any commercial or financial relationships that could be construed as a potential conflict of interest.
